# Background Risk Factors Associated with Shunt Intervention for Possible Idiopathic Normal Pressure Hydrocephalus: A Nationwide Hospital-Based Survey in Japan

**DOI:** 10.3233/JAD-180955

**Published:** 2019-03-29

**Authors:** Madoka Nakajima, Nagato Kuriyama, Masakazu Miyajima, Ikuko Ogino, Chihiro Akiba, Kaito Kawamura, Michiko Kurosawa, Yoshiyuki Watanabe, Wakaba Fukushima, Etsuro Mori, Takeo Kato, Hidenori Sugano, Yuichi Tange, Kostadin Karagiozov, Hajime Arai

**Affiliations:** a Department of Neurosurgery, Juntendo University Faculty of Medicine, Tokyo, Japan; b Department of Epidemiology for Community Health and Medicine, Graduate School of Medical Science, Kyoto Prefectural University of Medicine, Kyoto, Japan; c Department of Epidemiology and Environmental Health, Juntendo University Faculty of Medicine, Tokyo, Japan; d Department of Public Administrative Science for Community Health, Medicine and Welfare, Kyoto Prefectural University of Medicine Graduate School of Medical Science, Kyoto, Japan; e Department of Public Health, Osaka City University Graduate School of Medicine, Osaka, Japan; f Department of Psychiatry, Osaka University Graduate School of Medicine, Osaka, Japan; g Department of Neurology, Hematology, Metabolism, Endocrinology, and Diabetology, Faculty of Medicine, Yamagata University, Yamagata, Japan

**Keywords:** Alzheimer’s disease, cerebrospinal fluid shunt, geriatric care, healthy life expectancy, normal pressure hydrocephalus

## Abstract

**Background:**

Patients with idiopathic normal-pressure hydrocephalus (iNPH) are typically older adults with multiple comorbidities that are associated with a reduction in the efficacy of iNPH treatment via cerebrospinal fluid (CSF) shunt placement.

**Objective:**

The present study aimed to investigate the effectiveness of CSF shunt for iNPH using data from a nationwide epidemiological survey in Japan.

**Methods:**

We examined 1,423 patients (581 women) aged ≥60 years (median age [25%–75%]: 77 [73–80] years) who were diagnosed with iNPH following a hospital visit in 2012. Patients who experienced an improvement of at least one modified Rankin Scale (mRS) grade after the CSF shunt were classified as “improvement” while the remaining patients were classified as “non-improvement.” The efficacy of the shunt intervention (*n* = 842) was analyzed using a binomial logistic regression analysis.

**Results:**

An analysis of risk factors associated with shunt placement in patients with mRS grade 2 revealed an association between comorbid chronic ischemic lesions (odds ratio [OR], 2.28; 95% confidence interval [CI], 1.11–4.67; *p* = 0.025) and cervical spondylosis (OR, 3.62; 95% CI, 1.15–11.34; *p* = 0.027). Patients with mRS grade 3 at study entry had an association with comorbid Alzheimer’s disease (OR, 3.02; 95% CI, 1.44–6.31; *p* = 0.003).

**Conclusions:**

The results presented here showed that any age-related risk is minimal and should not be cause for rejection of surgical treatment options. Clinical decisions regarding CSF shunt should be individualized to each patient, with adequate consideration of the relative risks and benefits, including maximizing a healthy life expectancy.

## INTRODUCTION

Idiopathic normal-pressure hydrocephalus (iNPH) has been found to be particularly prevalent among older adults and is characterized by gait disturbances, cognitive impairments, and urinary incontinence [1, 2]. iNPH is thought to stem from difficulties with the clearance of waste metabolites from the brain [3–5]. Therefore, cerebrospinal fluid (CSF) shunts may have a certain degree of efficacy on iNPH [6, 7]. Guidelines for the treatment and diagnosis of iNPH have been established in US, EU, and Japan in recent years, which has improved outcomes [8–11]. However, iNPH patients are often older adults who experience comorbid disorders. These comorbidities can influence the efficacy of shunt treatment for iNPH; however, the risks that they present have not been specifically clarified in iNPH guidelines. Performing invasive procedures in elderly patients demands particular caution. Practitioners should be aware of the various risk factors involved in each case before creating a treatment and procedure plan.

Here, we report our findings on the efficacy of CSF shunt treatment in patients previously diagnosed with iNPH in a 2012 study [12].

## MATERIALS AND METHODS

### Patients and methods used in the nationwide epidemiological survey

In this study, patient information was collected using a questionnaire. We analyzed this information to determine which pre-operative background factors were most associated with improvements in post-operative symptomatology. We sought to determine the most important factors to consider when deciding whether a CSF shunt placement procedure is appropriate for a particular patient. iNPH patient symptomatology was assessed using a modified version of the Rankin Scale (mRS), which measures the degree of autonomy in activities of daily life [13]. An improvement of 1 point (from pre- to post-CSF shunt) or more on this scale was considered an indication that the shunt was effective.

The current study consisted of two sequential epidemiological surveys. First, we conducted a primary survey in patients with a diagnosis of iNPH who received medical care during 2012. Next, we conducted a secondary survey to clarify the clinical characteristics and treatment outcomes of these patients. This study was an extension of a previous nationwide epidemiological survey of iNPH cases [14]. Departments were selected from a specific list of medical institutions and hospitals for second surveys via methods standardized by the Research Committee on Epidemiology of Intractable Diseases in Japan, following the methods used for previous nationwide surveys for other diseases [15, 16]. The departments that were eligible for the survey were randomly extracted (per clinical unit) from a nationwide hospital database after each had been stratified on the basis of the hospital bed capacity as follows: hospitals attached to university schools of medicine (medical universities): 100%, general hospitals with ≥500 beds: 100%; 400–499 beds: 80%; 300–399 beds: 40%; 200–299 beds: 20%, 100–199 beds: 10%;≤99 beds: 5%; and special hospitals where there are high proportions of specific types of older adults patients (special-ranking hospitals): 100%. Extraction was performed using a stratified random sampling method, and the overall extraction rate was approximately 20%.

A nation-wide survey was conducted on patients that 1) were 60 years or older, 2) presented with enlargement of the brain ventricles, and 3) had one or more of the following symptoms: gait disturbances, cognitive impairments, or urinary incontinence. These were considered to be cases of possible iNPH, per Japanese guidelines for the diagnosis and treatment of iNPH [10, 17]. This survey was a primary survey to patients with a diagnosis of iNPH who also received medical care during 2012. Next, we administered a secondary survey to clarify the clinical characteristics and treatment outcomes in these cases. In addition, we separately asked the attending, treating physicians to confirm specific clinical details via a posted questionnaire (Supplementary Table 1). Using the survey data, we analyzed the risk associated with various factors in surgically-treated patients with iNPH in Japan.

In the first survey, 4,220 of a total 14,089 hospitals (459 university hospitals, 13,582 general hospitals, and 48 special stratified hospitals) were extracted and an epidemiological investigation was carried out by mail. In the primary survey, responses were obtained from 1,804 clinical departments (recovery rate: 42.7%); 3,079 patients met the diagnostic criteria for iNPH per Japanese diagnostic guidelines.

**Fig.1:**
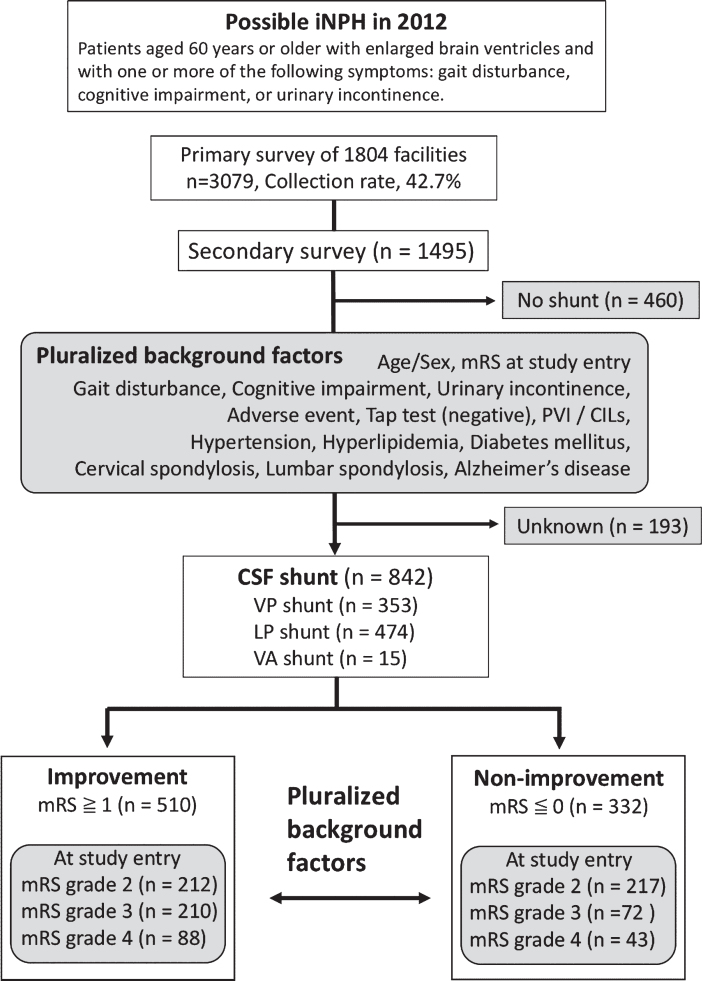
Study design flow chart. mRS, modified Rankin scale; CILs, chronic ischemic lesions; PVI, periventricular hyperintensity; iNPH, idiopathic normal-pressure hydrocephalus.

Of the 1,495 patients who were enrolled and completed both surveys, 193 had unknown mRS grades and 460 did not undergo shunt placement. These patients were excluded from the analyses (see the flow chart in Fig. 1). After exclusions, a total of 842 patients with possible iNPH (511 men and 331 women; median age (25%–75%), 77 (73–80) years) were included in the analyses (Table 1). These patients were divided into two groups according to surgical outcome: the “improvement” group included patients who showed at least a one-point improvement mRS grade after treatment. The “non-improvement” group included patients with no change or an increase in mRS grade after treatment.

**Table 1:** Baseline demographic data for patients diagnosed with idiopathic normal pressure hydrocephalus

	Shunt total	VP shunt	LP shunt	VA shunt
Total patients [Number]	842	353	474	15
Age, median (25%–75%)	77 (73–80)	76 (72–80)	77 (73–81)	77 (73–79)
Sex [Male number (%)]	511 (63.1%)	206 (58.4%)	295 (62.2%)	10 (66.7%)
mRS before shunt, median (25%–75%)	3 (2–3)	3 (2–3)	2 (2–3)	3 (3–4)
mRS after shunt, median (25%–75%)	2 (1–2)	2 (1–2)	2 (1–2)	2 (2–3)
mRS improvement [Number (%)]	518 (61.5%)	226 (64.0%)	283 (59.7%)	9 (60.0%)
Initial symptoms, number (%)
Gait disturbances	653 (77.6%)	277 (78.5%)	367 (77.4%)	9 (60.0%)
Cognitive impairments	296 (38.0%)	124 (35.1%)	166 (35.0%)	6 (40.0%)
Urinary incontinence	152 (20.2%)	60 (17.0%)	92 (19.4%)	0 (0%)
Comorbidity, number (%)
PVI	489 (54.9%)	221 (62.6%)	267 (56.3%)	1 (6.7%)
CILs	78 (9.3%)	37 (10.5%)	41 (8.6%)	0 (0%)
Hypertension	357 (44.5%)	146 (41.4%)	204 (43.0%)	7 (46.7%)
Hyperlipidemia	123 (15.3%)	48 (13.6%)	72 (15.2%)	3 (20.0%)
Diabetes mellitus	156 (18.6%)	62 (17.6%)	90 (19.0%)	4 (26.7%)
Cervical spondylosis	31 (3.5%)	16 (3.8%)	15 (3.1%)	0 (0%)
Lumbar spondylosis	114 (11.3%)	58 (13.9%)	50 (9.3%)	6 (35.3%)
Alzheimer’s disease	108 (13.4%)	38 (9.1%)	67 (12.4%)	3 (17.6%)

mRS, modified Rankin scale; PVI, periventricular hyperintensity; CILs, chronic ischemic lesions; VP, ventriculoperitoneal; LP, lumboperitoneal; VA, ventriculo-atrial.

### Questionnaire analysis

In the secondary survey, questionnaires were administered in all hospitals with reported cases of iNPH (Supplementary Table 1). These questionnaires initially examined sex, age, diagnostic classification entry data, initial symptoms, and comorbidities of patients with iNPH. In addition, physicians were asked to describe any cranial and spinal MRI findings. These included the presence/absence of ventricular dilatation; presence/absence of chronic ischemic lesions (CILs) ≤1.5 cm; presence/absence of white matter lesions directly below the cortex; and periventricular hyperintensity (PVI). Spinal cord MRI findings included the presence/absence of degenerative spondylosis in the cervical and lumbar spine. Lumbar CSF data were also included, because the survey asked whether CSF tap and drainage testing had been performed and if yes, what relevant outcomes were noted. Treatment information included the shunting method and system, complications, and ultimate clinical outcome. Therapeutic efficacy was evaluated based on both the attending physician’s assessment as well as mRS grade, which is an indicator of the patient’s ability to complete activities of daily living. To calculate the mRS grade, we modified the categories of the activities of daily living questions in the secondary survey card to the following: “Able to walk normally” = 1, “Able to walk alone while still handicapped” or “unable to perform all previous activities but able to take care for him/herself without assistance” = 2, “Able to walk only with a cane” or “requires some help, but able to walk without assistance” = 3, and “Wheelchair-bound” = 4.

Background factors including pre-intervention mRS grade, age, sex, initial symptom of gait disturbances, cognitive dysfunction, urinary disturbances, adverse events, tap test results, presence of CILs and/or PVI on imaging, and comorbidities including hypertension, hyperlipidemia, diabetes mellitus, cervical spondylosis, lumbar spondylosis, and Alzheimer’s disease.

### Statistical analyses

The analyses involved identifying confounding factors in both groups (non-improvement and improvement groups), followed by examining subjects in the CSF shunt treatment group to analyze factors that could influence changes in mRS grade. These factors were analyzed for each grade because the ratio of these factors showed great variability for each mRS grade at study entry (Fig. 1). To investigate the differences between the non-improvement and improvement groups, the Pearson chi-squared and Mann-Whitney U tests were used to analyze categorical variables.

A separate analysis of the associations between other factors and poor prognosis was conducted using a multiple logistic-regression. The significance level was set at <0.05 (two-tailed). All statistical analyses were performed using IBM SPSS ver. 22 (IBM Corp., Armonk, NY, USA).

### Ethical review

Patient consent was neither required nor sought, as this study was conducted in compliance with the ethical guidelines for epidemiological research (Notification No. 1 by the Ministry of Education, Culture, Sports and the Ministry of Health, Labor and Welfare in 2007). Any identifying data were protected in accordance with all relevant guidelines. The study was approved by the ethical committees of our institute.

## RESULTS

### Baseline characteristics

**Fig.2:**
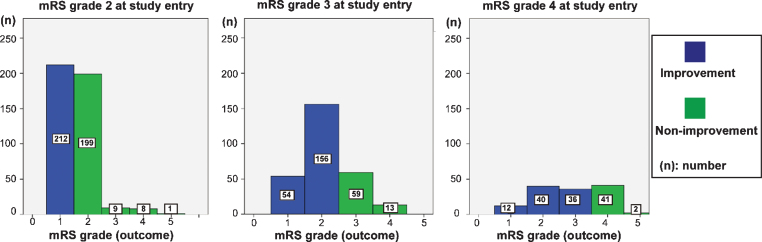
Outcome of each mRS grade at study entry. In mRS grade 2 at study entry, 212 showed an improved mRS grade, 199 showed maintenance of mRS grade, and 18 showed a worsening of mRS grade after CSF shunt. In patients with mRS grade 3 at study entry, 210 showed an improved mRS grade, 59 showed maintenance of mRS grade, and 13 showed a worsening of mRS grade after CSF shunt. In patients with mRS grade 4 at study entry, 88 showed an improved mRS grade, 41 showed maintenance of mRS grade, and 2 showed worsening of mRS grade.

Patients with iNPH who underwent shunt placement included ventriculoperitoneal (VP) (*n* = 353), lumboperitoneal (LP) (*n* = 474), and ventriculo-atrial (VA) shunts (*n* = 15, Table 1). There were no statistically significant differences between these shunt types and patient prognoses. Characteristics of the study population prior to shunt surgery are presented in Table 2. We divided patients who were diagnosed with iNPH and underwent CSF shunt treatment into improvement and non-improvement groups based on their mRS grade (Fig. 2). Next, we compared the background factors between the groups for each mRS grade. In the non-improvement group, there were significantly more patients with CILs and mRS grade 2 at study entry (*p* = 0.021) and Alzheimer’s disease and mRS grade 3 at study entry (*p* = 0.002). In the improvement group, there were significantly more patients with hyperlipidemia and mRS grade 2 at study entry (*p* = 0.034); hypertension and mRS grade 3 at study entry (*p* = 0.020); and diabetes mellitus and mRS grade 4 at study entry (*p* = 0.042). Factors that were not associated with a significant improvement in mRS grade after shunt treatment, were omitted from the risk factor analysis.

**Table 2:** Comparison between improved and non-improved mRS cases of background factors in patients who underwent shunt treatment

	mRS grade 2 at study entry, *n* = 429			mRS grade 3 at study entry, *n* = 282			mRS grade 4 at study entry, *n* = 131		
	improvement	non-improvement	*p*	improvement	non-improvement	*p*	improvement	non-improvement	*p*
Number (%)	212 (49.4%)	217 (50.6%)		210 (74.5%)	72 (25.5%)		88 (67.2%)	43 (32.8%)
Sex, Male	147 (68.7%)	146 (67.9%)	0.802	129 (60.2%)	37 (61.4%)	0.135	33 (37.5%)	19 (44.2%)	0.463
Age, median (25%–75%)	75 (71–79)	76 (72–80.5)	0.061	77 (73–80)	79.5 (74–81)	0.093	78 (75–81)	79 (75–83)	0.385
Initial symptoms
Gait disturbance	166 (78.3%)	158 (72.8%)	0.186	170 (81.9%)	61 (84.7%)	0.473	68 (77.3%)	30 (69.8%)	0.353
Cognitive impairment	69 (32.5%)	77 (35.5%)	0.521	68 (32.4%)	20 (30.6%)	0.467	38 (43.2%)	24 (55.8%)	0.174
Urinary incontinence	32 (15.1%)	33 (15.2%)	0.974	38 (18.1%)	14 (19.4%)	0.799	20 (22.7%)	15 (34.9%)	0.140
Tap test negative	10 (4.7%)	18 (8.3%)	0.134	12 (5.7%)	4 (5.6%)	0.960	6 (6.8%)	5 (11.6%)	0.351
Comorbidity
Adverse event	24 (11.3%)	26 (12.0%)	0.831	27 (12.9%)	12 (16.7%)	0.419	7 (8.0%)	4 (9.3%)	0.794
PVI	108 (50.9%)	122 (56.2%)	0.273	128 (61.0%)	45 (62.5%)	0.816	57 (62.9%)	29 (67.6%)	0.763
CILs	12 (5.7%)	26 (12.0%)	^*^0.021	18 (8.6%)	7 (9.7%)	0.767	11 (13.4%)	4 (5.4%)	0.589
Hypertension	898 (41.5%)	100 (46.1%)	0.340	97 (46.2%)	22 (30.6%)	^*^0.020	35 (39.8%)	15 (34.9%)	0.589
Hyperlipidemia	40 (18.9%)	25 (11.5%)	^*^0.034	32 (15.2%)	12 (16.7%)	0.773	11 (12.5%)	3 (7.0%)	0.337
Diabetes mellitus	40 (18.9%)	40 (18.4%)	0.908	49 (23.3%)	14 (19.4%)	0.494	12 (13.6%)	1 (2.3%)	^*^0.042
Cervical spondylosis	5 (2.3%)	12 (5.6%)	0.085	9 (4.3%)	1 (1.4%)	0.251	3 (3.4%)	1 (2.3%)	0.735
Lumbar spondylosis	20 (9.4%)	26 (12.0%)	0.394	26 (12.4%)	8 (11.1%)	0.775	12 (13.6%)	6 (14.0%)	0.961
Alzheimer disease	13 (6.1%)	24 (11.1%)	0.069	18 (8.6%)	16 (22.2%)	^**^0.002	13 (14.8%)	7 (16.3%)	0.822
Outcome
mRS (outcome), median (25%–75%)	1 (1–1)	2 (2–2)	^***^<0.001	2 (1–2)	3 (3–3)	^***^<0.001	2 (2–3)	4 (4–4)	^***^<0.001

^*^p < 0.05, ^**^p < 0.01, ^***^p < 0.001. mRS, modified Rankin scale; CILs, chronic ischemic lesions; PVI, periventricular hyperintensity.

### Prognostic investigations

**Fig.3:**
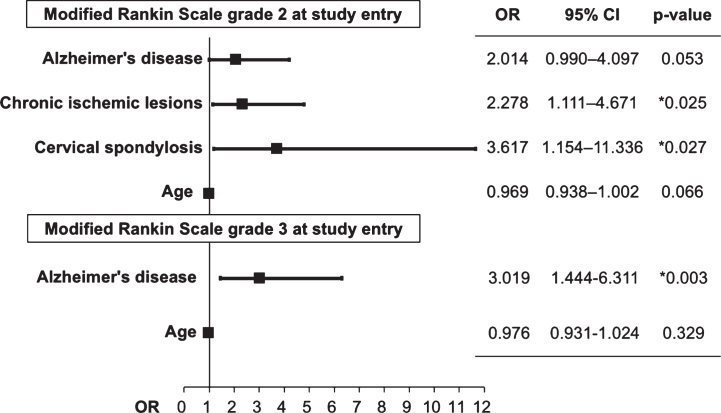
Odds ratio of predictive factors for mRS grade improvement following shunt intervention. OD, odds ratio; CI, confidence interval.

An analysis of risk factors associated with shunt placement in patients with mRS grade 2 at entry revealed an association between comorbid CILs (odds ratio [OR], 2.28; 95% confidence interval [CI], 1.11–4.67; *p* = 0.025) and cervical spondylosis (OR, 3.62; 95% CI, 1.15–11.34; *p* = 0.027) and between mRS grade 3 at study entry and comorbid Alzheimer’s disease (OR, 3.02; 95% CI, 1.44–6.31; *p* = 0.003) (Fig. 3).

## DISCUSSION

The present study revealed that clinical outcomes, as measured by mRS grade improvements, following CSF shunt were associated with comorbid CILs and cervical spondylosis in patients with mRS grade 2 at study entry and with comorbid Alzheimer’s disease in patients with mRS grade 3 at study entry. These background risk factors affected patient prognosis, while age, sex, a negative tap test, initial symptom of gait disturbance, cognitive impairment, urinary incontinence, and lumbar spine degeneration were not associated with significant differences in mRS grade.

### Cerebrovascular disease

The present study found that cerebral infarction did not affect the efficacy of CSF shunt; however, patients who exhibited CILs and mRS grade 2 at study entry had increased risk for poor prognosis. In line with this, previous authors have suggested that there is a cerebrovascular component to iNPH pathophysiology due to its strong association with vascular risk factors, such as hypertension [18, 19]. Hypertension causes arterial wall thickening and arteriosclerosis, predisposing patients to microinfarcts in vessels, including the lenticulostriate arteries, which traverse the brain parenchyma [20]. In addition, vascular injury in patients with iNPH may result in subsequent reductions in CSF turnover that impair the clearance of neurotoxic metabolites, such as amyloid-β and tau proteins [21]. However, CSF dynamics alone cannot explain all the features of iNPH.

Earlier studies have reported a good shunt response in patients with iNPH who exhibit extensive ischemic white matter lesions [22, 23]; however, radiological signs of cerebrovascular disease do not predict outcomes in these patients [24] and there is no difference in magnitude of improvements in patients with and without vascular comorbidities [6]. Interestingly, some studies have reported contrasting findings, with a reduction in improvement in patients with signs of ischemic cerebrovascular disease [25, 26].

Andrén reported that the presence of vascular comorbidities had no negative impact on outcomes, which were measured as improvements in mRS grade or subjective improvements in health condition, after 2–6 years. However, poorer mRS grade were noted after 6 years in patients with hypertension and a history of stroke [27]. The present report concluded that poorer outcomes among these patients indicate that they should not be excluded from CSF shunt, because vascular comorbidity showed only minor effects on the long-term outcomes in patients with iNPH.

### Cervical spondylosis

The present study found that cervical spondylosis did not affect the effectiveness of shunt treatment overall except in patients with mRS grade 2 at study entry. However, patients with iNPH are typically elderly with a high incidence of spinal disease complications; therefore, special attention is necessary in these patients if CSF shunt is the best course of action. Malm et al. have stated that if symptoms are progressive, the treatment of cervical spondylotic myelopathy should be prioritized over testing for iNPH or CSF shunt. Furthermore, they argue that patients with coexisting iNPH and lumbar canal stenosis should undergo CSF shunt. Notable exceptions to these guidelines are patients with pronounced neurological symptoms due to stenosis [28].

### Alzheimer’s disease

Alzheimer’s disease is the most common cause of dementia and a common comorbidity in patients with iNPH [29]. As expected, we observed a 15% comorbidity rate between the two conditions in the present study [14]. At shunt for patients with iNPH, the proportion of coexisting Alzheimer’s disease overall 8.6% in mRS grade 2, 12.1% in mRS grade 3, and 15.3% in mRS grade 4. The aggravation of mRS grade increased with age, as more patients with Alzheimer’s disease coexisted. Interestingly, comorbid Alzheimer’s disease affected iNPH patients’ shunt prognoses; comorbid Alzheimer’s disease was strongly correlated with prognosis in patients with a preoperative mRS grade 3. In the case of surgical intervention mRS grade 2, the risk of comorbidity with Alzheimer’s disease was not statistically significant, but marginal. It could still be a risk factor that affects prognosis.

Based on the data presented here, we advise caution when treating patients with comorbid Alzheimer’s disease. Comorbid Alzheimer’s disease affected patient prognosis following iNPH treatment [30, 31]; therefore, shunting significantly improved mRS grades in patients with comorbid Alzheimer’s disease. Given these results, shunt treatment should be considered even in patients with comorbidities such as Alzheimer’s disease, as it can improve their ability to perform activities of daily living.

Common reasons cited for avoiding surgery in elderly patients include age-related increases in surgical risk and a high association between spinal disease and cognitive, neurodegenerative disorders such as Alzheimer’s disease [28]. Surgical treatment may become less feasible with age, with an elevated risk of CSF shunt among elderly patients. However, the results presented here showed that any age-related risk is minimal and should not be cause for rejection of CSF shunt options. We argue that decisions regarding CSF shunt should be individualized to each patient, with proper consideration of the relative risks and benefits for each case, including healthy life expectancy.

The present study has several limitations worthy of consideration. First, this study involved a survey-based design that may have been subject to recall bias. Second, we did not assess the time from diagnosis to treatment or its effect on surgical outcomes. Analysis of this may have allowed for a more nuanced view of surgical outcomes based on presurgical patient status. Third, the present study employed a short follow-up period. While this allowed for more expediency, it limits the scope of our ability to assess postsurgical outcomes over time. These limitations should be considered when future studies are designed to confirm and/or extend the present study’s findings.

To the best of our knowledge, this is the first hospital-based survey study of patient background risk factors associated with shunt treatment outcomes in Japanese patients with iNPH. Comorbid Alzheimer’s disease in patients with mRS grade 3 at study entry; and CILs and cervical spondylosis in patients with mRS grade 2 at study entry had statistically significant effects on patient outcomes, as measured by post-operative mRS grade. Clinical decisions regarding shunt placement surgery should be individualized to each patient, with adequate consideration of the relative risks and benefits, including maximizing a healthy life expectancy.

## Supplementary Material

Supplementary Table
