# Supplementary material for: Background Risk Factors Associated with Shunt Intervention for Possible Idiopathic Normal Pressure Hydrocephalus: A Nationwide Hospital-Based Survey in Japan
Source: J Alzheimers Dis. 2019 Mar 29;68(2):735–44. doi: 10.3233/JAD-180955 (PMC6484254; doi:10.3233/JAD-180955)
Supplement: Supplementary Table [file jad-68-jad180955-s001.docx]

**Supplementary Material**

**Background Risk Factors Associated with Shunt Intervention for Possible Idiopathic Normal Pressure Hydrocephalus: A Nationwide Hospital-Based Survey in Japan**

**Supplementary Table 1.** Secondary survey card

| Sex & age | 1. man 2. woman  Date of birth　　Current age (　　)yo  Prefecture (　) City, ward, street (　　) | | | |
| --- | --- | --- | --- | --- |
| address |  |  |  |  |
| Diagnostic classification | 1. Possible iNPH  2. Probable iNPH  3. Definite iNPH  4. Possible iNPH with MRI support  5. Other | | | |
| Estimated time of onset (year and month) | ( )Year ( ) Month ( ) yo | | | |
| Diagnosis (year and month) | ( )Year ( ) Month ( ) yo | | | |
| Time of shunt treatment (year and month) | 1. ( )Year ( ) Month ( ) yo  2. Not subjected to shunt treatment | | | |
| Status of treatment | 1. Mainly as a hospitalized patient,  2. Mainly as an outpatient,  3. Hospitalization and outpatient treatment → Date of the last hospital visit  4. Transferred to another hospital (Name of the hospital where the patient was transferred:　　)  5. dead (year /month /yo) Was autopsy carried out? What was the direct cause of death?  6. Others  7. Unknown | | | |
| Onset of disease in consanguineous family members or housemates | 1. No  2. Relationship (a. Father, b. Mother, c. Elder brother, d. Younger brother, e. Elder sister, f. Younger sister, g. Child, h. Other)  3. Unknown | | | |
| Initial symptoms | 1.Gait Disturbance,  2.Cognitive Dysfunction,  3.Urinary Disturbance,  4. psychological symptom,  5. Other | | | |
| Complication | 1. Hypertension,  2. Hyperlipidemia,  3. Diabetes,  4. Cervical spondylosis,  5. lumbar spine degeneration,  6. Alzheimer Disease,  7. Tumor,  8. Others | | | |
| Clinical symptoms (during the entire course of the disease) | 1. Gait disturbance | | 1. No,  2. Yes (mild),  3. Yes (severe),  4. Unknown | |
|  | 1-1 Decrease in stride length | | 1. No,  2. Yes (mild),  3. Yes (severe),  4. Unknown | |
|  | 1-2 Decreased foot elevation | | 1. No,  2. Yes (Mild),  3. Yes (Severe),  4. Unknown | |
|  | 1-3 Wide-based gait | | 1. No,  2. Yes (Mild),  3. Yes (Severe),  4. Unknown | |
|  | 2. Cognitive disturbance | | 1. No,  2. Yes (Mild),  3. Yes (Severe),  4. Unknown | |
|  | 3. Urinary Disturbance | | 1. No,  2. Yes (Mild),  3. Yes (Severe),  4. Unknown | |
|  | 4. psychological symptom | | 1. No,  2. Yes (Mild),  3. Yes (Severe),  4. Unknown | |
|  | 5. Other motor disorders | | 1. No,  2. Yes,  3. Unknown | |
|  | 6. Muscular hypertonicity | | 1. No,  2. Yes (Mild),  3. Yes (Severe),  4. Unknown | |
| Imaging test findings | 1.Head CT abnormalities | | 1. No,  2. Yes,  3. Untested,  4. Unknown | |
|  | 2. Head MRI abnormalities | a. No  b. Yes →  c. Untested  d. Unknown | Ventricle dilatation | 1. No,  2. Yes, Evans Index  *a*. Less than 0.3, *b*. 0.3 or higher.  Lesions around the lateral ventricles.  [a] Yes, [b] No.  Ischemic lesions with a radius of 1.5cm or larger  [a] Yes, [b] No.  Lesion in the white matter directly under the cortex. *a*. No, *b*. Yes  3. Unknown |
|  | 3. Spinal MRI abnormalities | a. No  b. Yes →  c. Untested  d. Unknown | Lesions in the cervical spine  Lesions in the lumbar spine | 1. No, 2. Yes  1. No, 2. Yes |
|  | 4．Cerebral blood flow scintigraphy | a. No  b. Yes →  c. Untested  d. Unknown | Decreased blood flow in the frontal lobe  Decreased blood flow around the corpus callosum  Decreased blood flow in the sylvian fissure | 1. No, 2. Yes  1. No, 2. Yes  1. No, 2. Yes |
| Laboratory findings | 1. Abnormal cerebrospinal fluid pressure | | 1. No,  2. Yes ( cmH_2_O),  3. Unknown | |
|  | 2. Elevated cell count in the cerebrospinal fluid | | 1. No,  2. Yes ( cmH_2_O),  3. Unknown | |
|  | 3. Elevated levels of proteins in the cerebrospinal fluid  4. tap test | | 1. No,  2. Yes ( mm^3^),  3. Untested,  4. Unknown | |
|  |  | | 1. Negative,  2. Positive,  3. Untested,  4. Unknown | |
|  | 5. drainage test | | 1.Negative,  2. Positive,  3. Untested,  4. Unknown | |

Treatment

| CSF shunt | 1. No  2. Yes → Effectiveness of treatment (1. No; 2. Yes)  3. Unknown |
| --- | --- |
| Type of shunt | VP shunt  1. No  2. Yes → Effectiveness of treatment (1. No; 2. Yes; 3. Unknown)  3.Unknown |
|  | LP shunt  1. No  2. Yes → Effectiveness of treatment (1. No; 2. Yes; 3. Unknown)  3.Unknown |
|  | VA shunt  1. No  2. Yes → Effectiveness of treatment (1. No; 2. Yes; 3. Unknown)  3. Unknown |
| Shunt system | 1. Adjustable (Programmable) valve  2. Fixed valve  3. Other  4. Unknown |
| Complication | 1. Yes  2. No |

Outcome

| Activities of daily living at the initial visit | 1. Bedridden,  2. Wheelchair,  3. Able to walk only with a cane or support,  4. Able to walk by oneself while still handicapped,  5. Able to walk normally,  6. Dead,  7. Others |
| --- | --- |
| Activities of daily living at the last visit | 1. Bedridden,  2. Wheelchair,  3. "Able to walk only with a cane or support" or “requires some help, but able to walk without assistance”,  4. "Able to walk by oneself while still handicapped" or “unable to perform all previous activities but able to look after own affairs without assistance”,  5. Able to walk normally,  6. Death,  7. Others |
